# The Effect of Exertion on Heart Rate and Rating of Perceived Exertion in Acutely Concussed Individuals

**DOI:** 10.4172/2155-9562.1000388

**Published:** 2016-08-23

**Authors:** Andrea Hinds, John Leddy, Michael Freitas, Natalie Czuczman, Barry Willer

**Affiliations:** 1 Department of Psychiatry, University Sports Medicine, Buffalo New York, USA; 2 UBMD Orthopaedics and Sports Medicine, University at Buffalo, Buffalo, New York, USA; 3 SUNY Jacobs School of Medicine, Buffalo, New York, USA

**Keywords:** Cardiovascular, Concussion, Exercise, Autonomic, Heart rate, Exertion, Acute

## Abstract

**Objective:**

Research suggests that one physiological effect of concussion is a disruption in regulation of autonomic nervous system control that affects the balance between parasympathetic and sympathetic output. While changes in heart rate after concussion have been observed, the nature of the heart rate change during progressive exercise has not been well evaluated in acutely symptomatic patients. Additionally, little is known about the relationship between HR and RPE in this population.

**Methods:**

We compared changes in heart rate and perceived effort during graded treadmill exertion in recently concussed patients to elucidate the effect of brain injury on cardiovascular response to exercise. Resting HR, HR on exercise initiation, and changes in HR and RPE during the Buffalo Concussion Treadmill Test (BCTT) were compared on two test visits: When patients were symptomatic (acute) and after recovery. Results were compared with the test-retest results obtained from a control group consisting of healthy, non-concussed individuals.

**Results:**

Patients had a significantly lower HR at onset of exercise when acutely concussed as compared to when recovered and reported greater perceived exertion at every exercise intensity level when symptomatic, despite exercising at lower workloads, than when recovered. Sympathetic response to increased exertion was not affected by concussion - HR increased in response to exercise at a comparable rate in both tests. These differences observed in response to exercise between the first BCTT and follow-up evaluation in initially concussed patients were not present in non-concussed individuals.

**Conclusion:**

Our results suggest that during the acute phase after concussion, acutely concussed patients demonstrated an impaired ability to shift from parasympathetic to sympathetic control over heart rate at the onset of exercise. Changes in the autonomic nervous system after concussion may be more complex than previously reported. Continued evaluation of autonomic regulatory effects in the acute phase after concussion is warranted.

## Introduction

There is increasing interest about the effects of acute concussive injury on the capacity of the central nervous system to appropriately regulate peripheral functions such as cardiovascular rhythm, metabolism and temperature regulation and how this may relate to prolonged recovery from concussion [1–4]. Studies that have addressed disruption in regulatory function after mild or severe traumatic brain injury have generally focused on abnormalities in autonomic control over the sympathetic and parasympathetic nervous system, including control of heart rate (HR) and heart rate variability (HRV) [3,5–7]. Gall et al. [5,6] showed that HR after physical exertion was elevated in recently concussed athletes when compared with healthy controls, strongly suggesting that acute concussion shifts the balance in the autonomic nervous system toward hyperactivity of the sympathetic system. Elevated HR in response to stress or exercise would then be indicative of an uncoupling of the autonomic and cardiovascular systems and an inability of the parasympathetic system to sufficiently contribute to beat-to-beat variability [5]. Others have argued that autonomic dysregulation is a primary pathophysiological characteristic of mild traumatic brain injury. Leddy et al., for example, argued that concussion is directly associated with diffuse physiological and metabolic changes, including dysfunction of the regulation of cerebral blood flow (CBF) and uncoupling or distortion of cardiac autonomic control [3]. Studies examining HRV have focused on difficulties in HR recovery (the ability to re-engage the parasympathetic system after termination of cardiovascular exertion) and changes in the relative contribution of the parasympathetic nervous system to the cardiac rhythm following injury [8–10]. A recent animal study found some evidence to support these arguments, indicating changes in autonomic control over HR in rats forced to exercise after an induced concussion [7]. While research directly examining autonomic regulatory changes following concussion is limited, some support comes from the literature on severe traumatic brain injury (TBI). Significant changes in cardiac autonomic control after TBI have been reported, including parasympathetic withdrawal from the cardiac rhythm (resulting in severe cardiac abnormalities), orthostatic intolerance, and tachycardia [11,12].

Although some literature supports autonomic consequences of brain injury, there are discrepancies that make it difficult to generalize these findings. For instance, some studies suggest increases in parasympathetic influence (or lack of parasympathetic withdrawal) in the very early stages after acute injury [9,13]. Similarly, an animal study in 2013 by Griesbach et al. [7] found reduced resting HRs in rats on the day of injury. We became interested in elucidating the degree of autonomic dysregulation in individuals diagnosed with concussion by examining HR control upon initiating and engaging in an exercise task. Since previous studies on exercise have also looked at subjective measures of work load and its relation to actual HR during exercise, it makes sense to evaluate rating of perceived exertion (RPE) [14] during exercise in patients with concussion. Based on previous findings, we hypothesized that concussed individuals would demonstrate cardiovascular dysregulation indicative of abnormal autonomic control over the heart rhythm during engagement with exercise, and that physiological recovery of the autonomic nervous system would correlate with resolution of concussion symptoms in these individuals.

To comprehensively test the influence of concussion on autonomic function, we conducted a prospective, controlled evaluation of cardiovascular function in recently concussed patients.

## Methods

### Participants

We conducted an IRB-approved prospective study on patients visiting a concussion clinic over an 18-month period (2014–2016) for treatment of acute concussion. Recently concussed patients (n=40; mean time since injury, five days) were selected for the study if they were symptomatic (as defined by symptom load as reported on the Sport Concussion Assessment Tool, SCAT-2) [2] at the time of physician evaluation and provided informed consent. Symptom presentation was variable, with patients reporting, for example, headache and distortions in cognition and mood that were exacerbated by physical exertion. Patients were eligible if they did not have any additional conditions that would preclude them from exercising on the treadmill (such as orthopedic injury or cardiovascular illness). Mean age of participants was 15.5 years (age ranged from 12–18 years) and the study included both symptomatic male and female athletes (23 males and 17 females). Mean BMI for participants was 19.6. A sample of healthy, non-concussed athletes was run through the protocol as a control group. Athletes (n=30) were selected if they were asymptomatic for concussion and had not experienced a head injury during the previous six month period and could safely exert themselves to physical exhaustion. Mean age of control participants was 15.9 years (age ranged from 13–18 years) and the study included both males and females (18 males and 12 females). Mean BMI for participants was 18.6. Demographics for participant groups are provided in Table 1.

### Procedures

We evaluated HR and RPE changes during graded exercise on the Buffalo Concussion Treadmill Test (BCTT) [14] in patients experiencing post-concussive symptoms. The BCTT is a test of exercise tolerance that consists of graded exercise on a treadmill until the participant reaches maximum exertion or experiences an exacerbation of symptoms (i.e., demonstrates exercise intolerance) [15].

The BCTT has previously been demonstrated to have good inter-rater and test-retest reliability [15]. During the BCTT, exercise intensity (treadmill grade) is increased each minute until termination of the test. After each minute, participants indicated their RPE on a scale of 6–20 (the Borg scale) [14] and average HR each minute was calculated from recordings extracted from a Polar HR monitor (Polar Electro, Model FT1). Heart rate at rest and at the initiation of exercise (exercise time zero) were recorded to compare HR before and at the onset of exertion. Participants repeated the treadmill protocol when they reported being asymptomatic and were independently evaluated by a blinded physician as being recovered from concussion. Recovery was defined as resolution of symptoms, no symptom exacerbation during treadmill exertion, and a normal physical examination. This protocol allowed for each participant to act as his or her own control, allowing us to directly compare autonomic measures in the same participant during the acute and recovered phases. For the control group, the second BCTT was completed one week following the initial visit. For both groups, we looked at changes over the first nine minutes of graded exercise even though some subjects were able to exercise much longer, as this represented the average time to test completion.

Timing of the BCTT was controlled to minimize potential confounds from pre-exercise activity. Participants completed the test at approximately the same time of day at each visit after spending at least one hour in the medical clinic before testing. This reduced the possible influence of caffeine, other drugs, or physical or cognitive exertion immediately before testing. All participants were instructed to refrain from new medications between testing sessions, and to report any major changes to their daily lifestyle.

### Statistical Analyses

Resting HR and HR and RPE at each time point during exercise were compared between groups using two-sample t-tests. Age, body mass index (BMI), and resting HR were also compared between groups using two-sample t-tests. For both groups, two repeated-measures analysis of variance (RMANOVA) were performed to examine both the change in HR over time and changes in RPE over time, both as a factor of group (acute or control). Age was considered as a covariate in each analysis of HR change but did not contribute significantly to the model. Significance was defined as a p-value <0.05 and all statistical analyses were done using SPSS.

## Results

**Figure 1:**
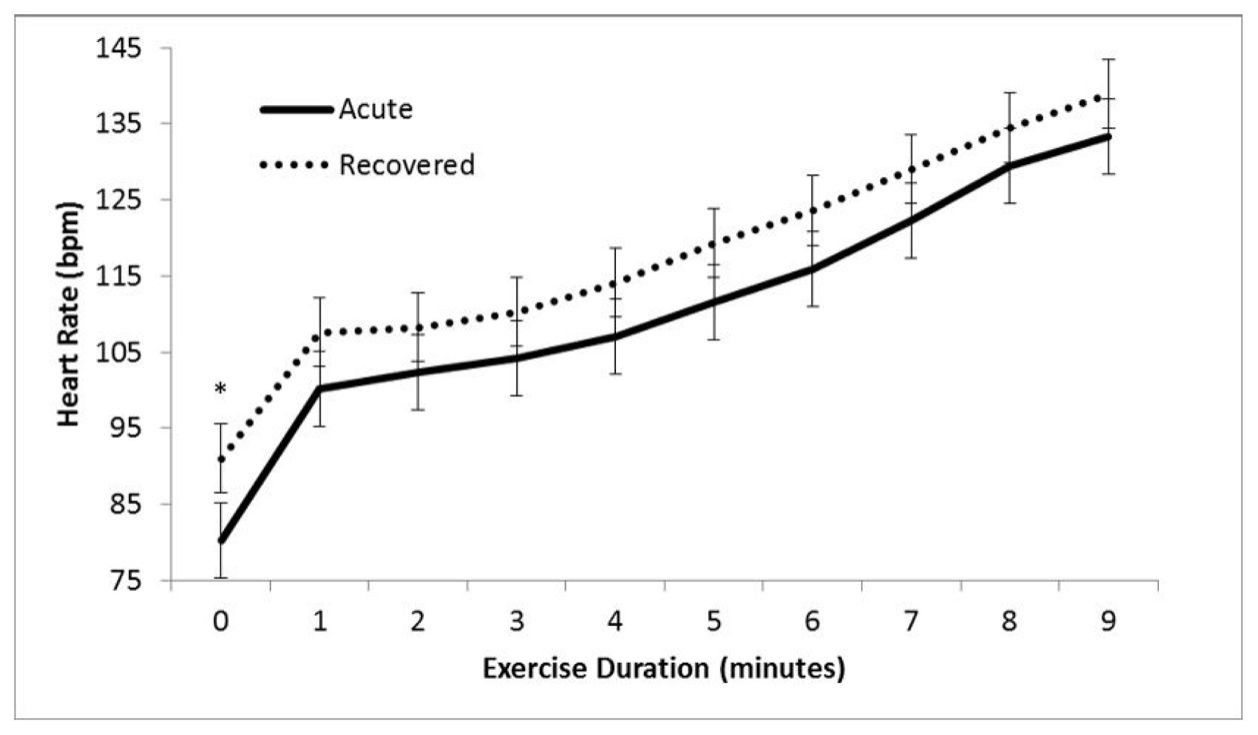
Change in heart rate with exercise in concussed patients. Average HR at each minute of exercise (when acutely symptomatic versus when recovered from concussion); n=40; * indicates p<0.05.

For acutely concussed patients, resting HR did not differ significantly when participants were symptomatic when compared with same participants after recovery (72 ± 6.3 vs. 74 ± 6.7 BPM, p>0.05). At the start of exercise (time 0), HR was significantly lower when participants were acutely concussed than when they were recovered (p=0.015); however, the relative increase in HR seen during each successive increase in exercise intensity (increased HR corresponding to an increase in exercise exertion) was not significantly different when patients were acutely concussed as compared to when they were recovered (Figure 1).

**Figure 2:**
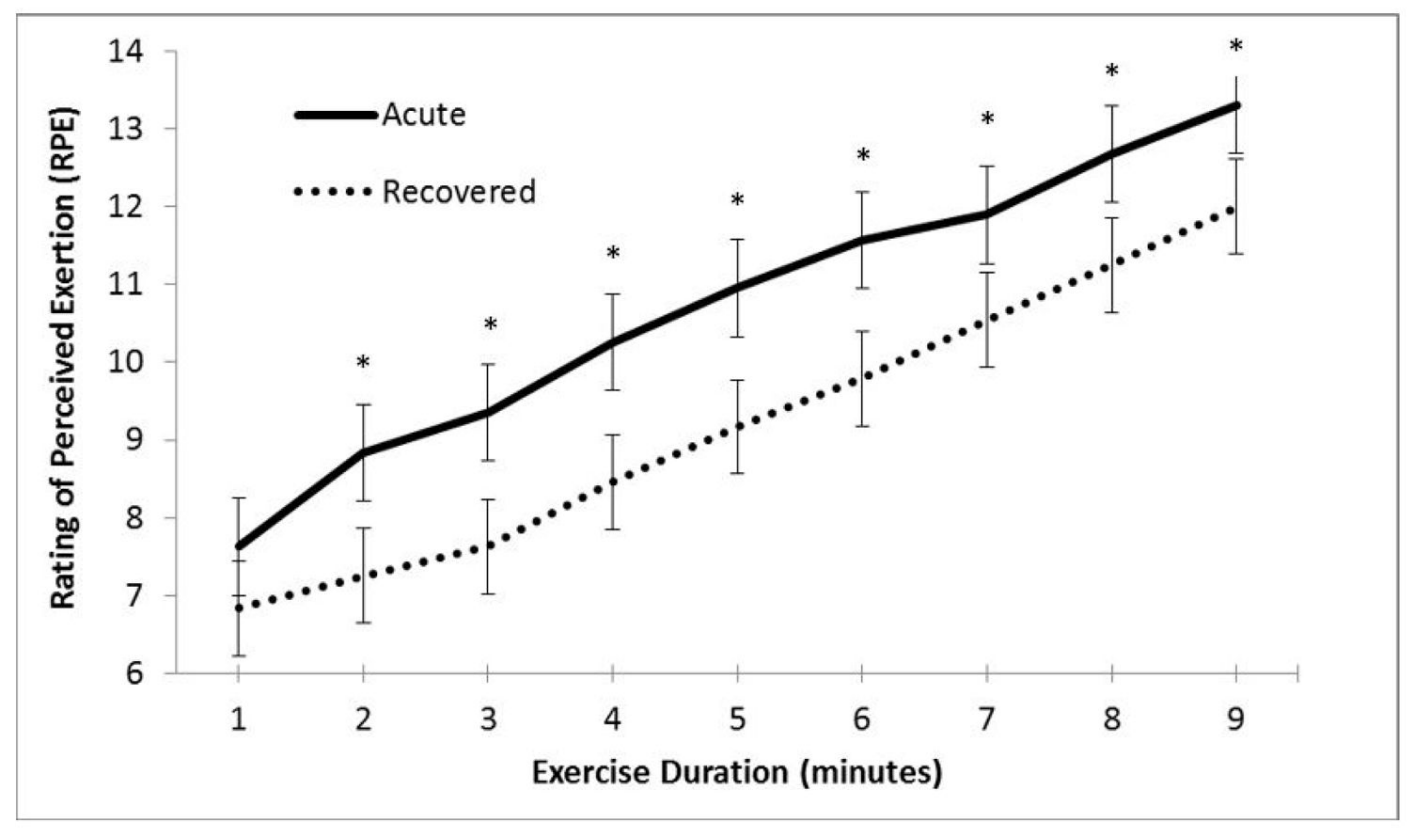
Change in RPE with exercise in concussed patients. Average RPE at each minute of exercise (when acutely symptomatic versus when recovered from concussion); n=40; * indicates p<0.05.

In contrast, the discrepancy between predicted and observed rating of exertion (RPE) was consistently higher when patients were acutely symptomatic. RPE was consistently rated higher for comparable work in symptomatic participants versus when they were recovered, despite relatively similar HRs during exertion and comparable level of physical work (p<0.01; Figure 2). Age and gender were not significant factors. This suggests that self-perception of exertion is skewed in the concussed individuals.

**Figure 3:**
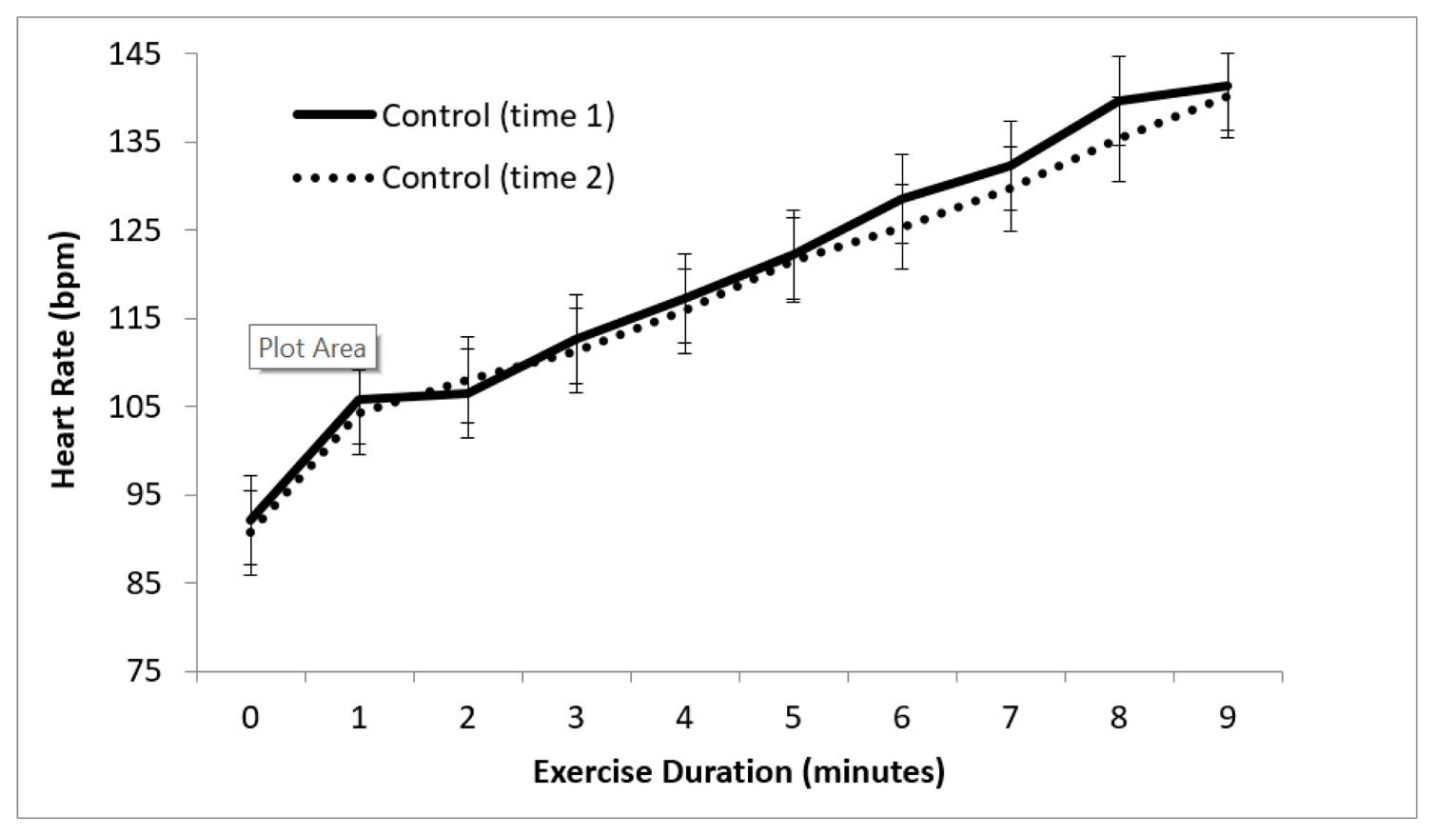
Change in heart rate with exercise in healthy controls. Average HR at each minute of exercise (controls at initial visit versus follow-up visit); n=30.

**Figure 4:**
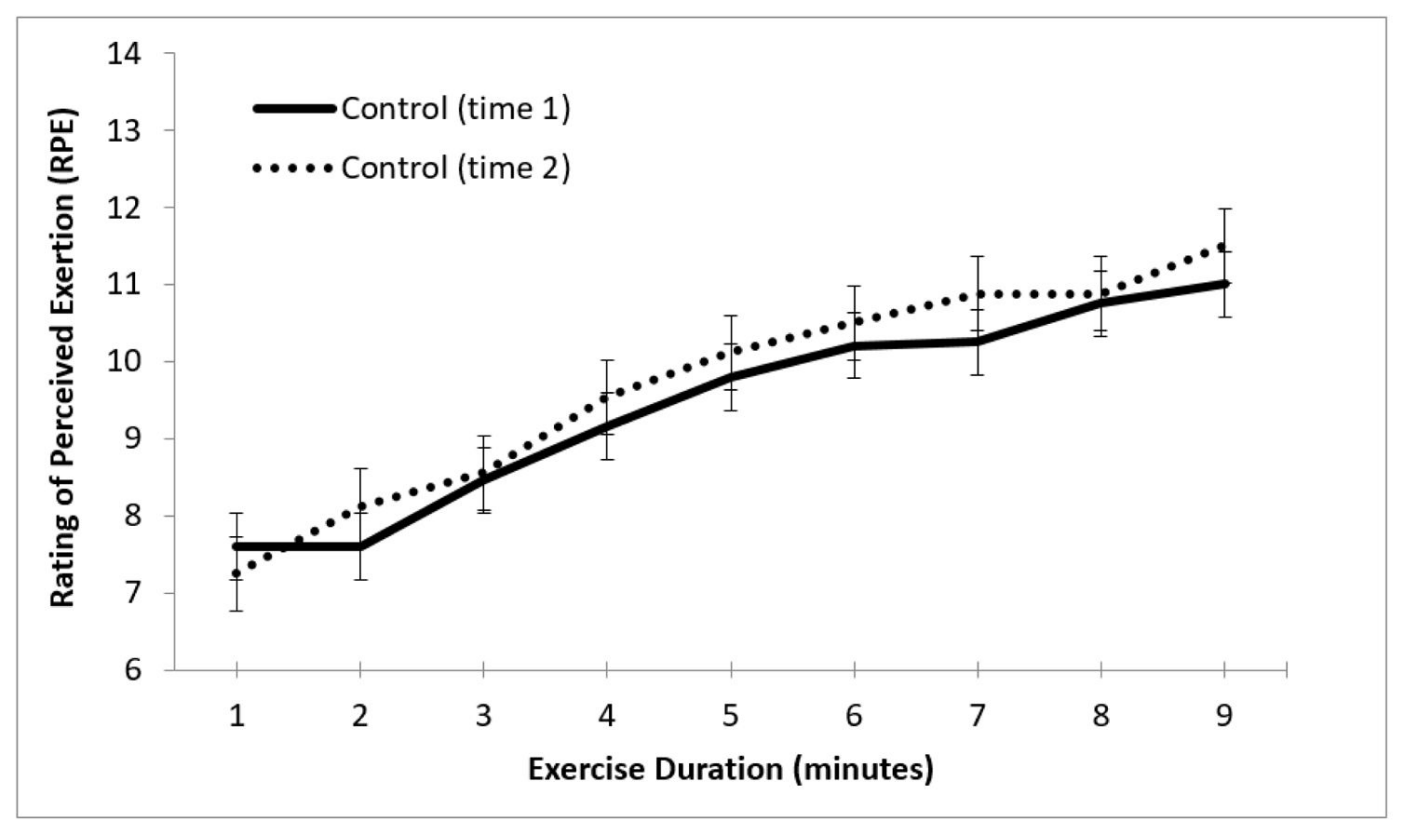
Change in RPE with exercise in healthy controls. Average RPE at each minute of exercise (controls at initial visit versus follow-up visit); n=30.

For control participants, there were no significant differences between HR at initiation of exercise or relative change in HR with exertion between visits (Figure 3), nor any differences in RPE change with exercise on repeated administration of the treadmill test (Figure 4).

## Discussion

To our knowledge, this is the first study to evaluate cardiac performance during progressive aerobic exercise in acutely symptomatic concussed individuals. The evaluation of cardiovascular response to exercise as measured during treadmill testing in patients with acute concussion symptoms revealed a significant reduction in HR at the point of exercise initiation when compared with the same patients at the time of recovery. While the typical HR rise seen with increasing workload [14] was observed regardless of concussion status, acutely symptomatic patients did not have an exaggerated cardiovascular response to physical exertion. This study allowed us to directly observe the influence of concussion on regulation of the cardiac rhythm in symptomatic athletes. Comparable research has limited the evaluation of regulatory dysfunction to patients already asymptomatic [5,6], during isometric exercise [10] or while the individual is at rest [13]. In addition, while other studies have explored the capacity of the autonomic system to adjust and respond to prolonged steady-state exercise, our study specifically evaluated the anticipatory response to exercise and adjustment to a change in exertion level over time, which is more representative of athletic performance. The preponderance of the evidence in the literature suggests that regulatory and physiological changes following concussion shift the balance away from parasympathetic influence on the heart toward sympathetic “overdrive” [5,7]. Our results, however, indicate that recently concussed individuals lack control over parasympathetic withdrawal of influence on cardiac rhythm, resulting in a dampened sympathetic response on initiation of exercise. This dampened autonomic response to physical activity has been described in studies of autonomic dysregulation in other clinical populations [16].

Careful consideration of the literature reveals subtle indices of a parasympathetic system that is slow to withdraw in cases of acute concussion. La Fountaine et al. [10] demonstrated that autonomic activity at rest could differentiate concussed from non-concussed controls and that vagal dysfunction after concussion resulted in an increase in vagal activity at 48 hours post-injury despite evidence for sympathetic hyperactivity one week and two weeks later [13]. Similarly, an animal model of brain injury showed a significantly lower resting HR in concussed rats on the day of injury when compared with sham-treated rats [7], suggesting that the nature of autonomic system dysfunction after injury is time dependent, with vagal dysfunction from a state of sluggish withdrawal to one of disordered engagement or re-engagement. Our results suggest that systematic testing of concussed athletes in the acute phase using progressive aerobic exercise with pre-determined stopping criteria can identify early regulatory problems that will be modified as the individual begins to recover. Despite an increase in sympathetic output in response to exertion, HR may be blunted in acutely concussed individuals by an excess of parasympathetic vagal tone. During recovery, however, this response then appears to shift to one of sympathetic hyperactivity as the appropriate parasympathetic-sympathetic balance is restored.

Regulatory abnormalities are likely multifaceted and many involve more subtle disturbance of autonomic pathways than previously suggested. A more sophisticated understanding of cardiovascular-autonomic dysregulation after concussive injury is imperative for the evaluation and treatment of concussion. Current therapies for concussed patients, including some which utilize biofeedback for retraining of the autonomic system after injury [1], while well-intentioned, may be limited by an oversimplification of the nature of this dysregulation. In contrast to the consideration of increased cardiac sympathetic activity after injury as the predominant cause of autonomic problems after concussion, our results suggest that earlier intervention to target the parasympathetic system may help prevent prolonged regulatory problems.

While this study provides evidence for autonomic dysregulation after acute concussion, it is limited in its lack of additional measures of autonomic function. Further investigation into the time-course of autonomic disturbance after injury is warranted and should include additional variables of interest such as blood pressure, temperature, heart rate variability, and cardiac output. In addition, the present study does not directly provide evidence for the viability of providing therapeutic interventions for recently concussed, symptomatic individuals that may directly target disruptions in autonomic function.

Future study into the benefit of rehabilitating the autonomic nervous system as a focus for recovery from concussion is warranted. Future research is also needed to determine if these results can be reliably extended to populations beyond our sample group of adolescent athletes.

## Figures and Tables

**Table 1:** Participant demographics.

	Acutely Concussed Patients	Non-Injured Controls
**Total N**	40	30
Days Since Concussion	5 ± 1.1	Not applicable
Mean Age at Testing	15.5 ± 0.9	15.9 ± 0.5
Mean BMI	19.6 ± 1.4	18.6 ± 1.9
Mean Resting Heart Rate (bpm)	72 ± 6.3	70 ± 7.1
**Gender Frequency**		
Females	17 (42.5%)	12 (40.0%)
Males	23 (57.5%)	18 (60.0%)
